# Treatment effects of N-acetyl cysteine on resting-state functional MRI and cognitive performance in patients with chronic mild traumatic brain injury: a longitudinal study

**DOI:** 10.3389/fneur.2024.1282198

**Published:** 2024-01-17

**Authors:** Faezeh Vedaei, Andrew B. Newberg, Mahdi Alizadeh, George Zabrecky, Emily Navarreto, Chloe Hriso, Nancy Wintering, Feroze B. Mohamed, Daniel Monti

**Affiliations:** ^1^Department of Radiology, Jefferson Integrated Magnetic Resonance Imaging Center, Thomas Jefferson University, Philadelphia, PA, United States; ^2^Department of Integrative Medicine and Nutritional Sciences, Marcus Institute of Integrative Health, Thomas Jefferson University, Philadelphia, PA, United States

**Keywords:** mild traumatic brain injury, resting-state fMRI, degree centrality, fractional amplitude of low frequency fluctuations, functional connectivity strength, longitudinal study, N-acetyl cysteine treatment

## Abstract

Mild traumatic brain injury (mTBI) is a significant public health concern, specially characterized by a complex pattern of abnormal neural activity and functional connectivity. It is often associated with a broad spectrum of short-term and long-term cognitive and behavioral symptoms including memory dysfunction, headache, and balance difficulties. Furthermore, there is evidence that oxidative stress significantly contributes to these symptoms and neurophysiological changes. The purpose of this study was to assess the effect of N-acetylcysteine (NAC) on brain function and chronic symptoms in mTBI patients. Fifty patients diagnosed with chronic mTBI participated in this study. They were categorized into two groups including controls (CN, *n* = 25), and patients receiving treatment with N-acetyl cysteine (NAC, *n* = 25). NAC group received 50 mg/kg intravenous (IV) medication once a day per week. In the rest of the week, they took one 500 mg NAC tablet twice per day. Each patient underwent rs-fMRI scanning at two timepoints including the baseline and 3 months later at follow-up, while the NAC group received a combination of oral and IV NAC over that time. Three rs-fMRI metrics were measured including fractional amplitude of low frequency fluctuations (fALFF), degree centrality (DC), and functional connectivity strength (FCS). Neuropsychological tests were also assessed at the same day of scanning for each patient. The alteration of rs-fMRI metrics and cognitive scores were measured over 3 months treatment with NAC. Then, the correlation analysis was executed to estimate the association of rs-fMRI measurements and cognitive performance over 3 months (*p* < 0.05). Two significant group-by-time effects demonstrated the changes of rs-fMRI metrics particularly in the regions located in the default mode network (DMN), sensorimotor network, and emotional circuits that were significantly correlated with cognitive function recovery over 3 months treatment with NAC (*p* < 0.05). NAC appears to modulate neural activity and functional connectivity in specific brain networks, and these changes could account for clinical improvement. This study confirmed the short-term therapeutic efficacy of NAC in chronic mTBI patients that may contribute to understanding of neurophysiological effects of NAC in mTBI. These findings encourage further research on long-term neurobehavioral assessment of NAC assisting development of therapeutic plans in mTBI.

## Introduction

Traumatic brain injury (TBI) is one of the causes of death and long-term disability worldwide. It has been estimated annually 1.7 million people in the United States and 10 million people around the world suffer a TBI (1, 2). The majority of TBIs are mild to moderate and account for 80–90% of the cases. Although the injury itself is regarded as mild, many of these patients are living with one or more disabilities from TBI in the subacute and chronic stages (3, 4). Individuals with TBI are prone to develop cognitive, behavioral, and sensorimotor impairments including attention deficit, memory loss, lower executive ability that necessitate long-term access to health care and disability services (5). Furthermore, from a clinical perspective, they are more likely to develop neurodegenerative disorders such as Alzheimer’s disease (AD) and Parkinson’s disease (PD) later in the lifetime (6, 7).

Mild traumatic brain injury (mTBI) is characterized by short-term and long-term clinical and cognitive dysfunction caused by brain function alterations that can be shown using advanced neuroimaging techniques including resting-state functional magnetic resonance imaging (rs-fMRI) (8). By measuring low frequency oscillations (LFOs) in the blood oxygenation level- dependent (BOLD) signal, rs-fMRI can reflect spontaneous brain neural activity within the range of 0.01–0.1 Hz that is assumed to be linked with neural fluctuations at rest.

The measurements including fractional amplitude of low frequency fluctuations (fALFF), degree centrality (DC), and functional connectivity strength (FCS) have been shown to provide robust biomarkers for depicting regional properties of rs-fMRI BOLD signal. fALFF is the fast Fourier transform (FFT)-based metric of LFO amplitude. It measures the relative contribution of the power of low-frequency BOLD signal fluctuations reflecting the magnitude of neural activity (9). Likewise, the DC is a graph theory-based rs-fMRI metric that reveals the core-hub architecture of brain networks. It considers each gray matter (GM) voxel as a node of the network and measures the total weight of connections for a given node, reflecting its role as a central hub in the integration of the global network (10). Also, FCS is a voxel-based rs-fMRI measurement estimating Pearson’s correlations between time series of any pairs of brain voxels that results in whole-brain FC matrix (11).

An increasing number of studies have reported disrupted brain functional characteristics using LFO-based rs-fMRI metrics in a wide range of neuropsychiatric and cognitive disorders including AD (12), PD (13), major depression and anxiety disorder (14), schizophrenia (15), and mTBI (16–18). While previous studies have extensively been cross-sectional in design, a growing body of longitudinal studies have shown brain function changes following therapeutic treatment plans and recovery from diseases (19–21). Several longitudinal studies obtained rs-fMRI imaging to investigate brain function alteration and cognitive recovery after mTBI. For instance, Madhavan et al. used rs-fMRI imaging in a longitudinal study of patients with mTBI over 3 months post-injury (22). They measured fALFF and seed-based functional connectivity (FC) over 3 months recovery and examined the association between rs-fMRI measurements and the symptom severity score (SSS). They have shown that increased fALFF as well as increased FC between brain networks including motor, default mode network (DMN), and visual network are associated with lower SSS that can be used as potential biomarkers for predicting mTBI recovery and clinical outcomes (22). Additionally, Palacios et al. in a longitudinal study in patients in semi-acute stage of mTBI examined FC of resting-state networks using independent component analysis (ICA) as well as its association with cognitive performance using neuropsychological tests over 6 months post-injury (23). They found that reduction of FC in several brain networks involved in behavioral process including the DMN, executive control network, frontoparietal network, dorsal attention network, and orbitofrontal network over 6 months post-injury that was associated with gradual, natural improvement of cognitive performance (23). However, no neuroimaging study has tracked brain function longitudinally including a treatment phase with medications or supplements in mTBI patients.

The pathophysiology of mTBI involves secondary injury-induced damage causing a cascade of molecular mechanisms evolving over time and assumed to be responsible for progressive and/or chronic neurological and cognitive impairment after trauma (24–26). Central nervous system (CNS) inflammation and oxidative stress are commonly believed to cause functional disruption and to contribute to post-injury cell death which may last many years in mTBI patients (27–29). Oxidative stress refers to an imbalance between the production of reactive oxygen species (ROS) and the ability of the body to detoxify them or repair the resulting damage. When a TBI occurs, it can trigger a cascade of events that lead to increased oxidative stress. This oxidative stress can damage cell membranes, proteins, and DNA, contributing to the progression of secondary brain injury. The brain is particularly vulnerable to oxidative stress due to its high metabolic rate, high oxygen consumption, and relatively low levels of antioxidant defenses. The hippocampus, a region of the brain involved in learning and memory, is particularly sensitive to oxidative stress. Studies have suggested that oxidative stress following TBI can contribute to memory impairment and cognitive dysfunction. Several incidents may contribute to oxidative stress and cognitive dysfunction following mTBI such as neuroinflammation, mitochondrial dysfunction, blood–brain barrio (BBB) disruption (30, 31).

N-acetyl cysteine (NAC) is an U.S. Food and Drug Administration (FDA) approved medication and has been known as an effective therapeutic approach after central and/or peripheral nervous system injury due to its antioxidant and anti-inflammatory properties (26, 29, 32). It is a neuroprotective agent and has beneficial effects on oxidative stress that induces diseases and clinical disorders and is well tolerated with low side effect profile making it an excellent candidate for treatment in clinical and preclinical TBI (33, 34). Several animal studies have shown the beneficial effects of NAC in neurobehavioral damage recovery induced by brain injury and suggested the potentially valuable role of NAC to treat TBI (26, 35–37). Also, recent human studies have confirmed the anti-inflammatory effects of NAC and its beneficial impact on deceleration of neural damage and neurodegenerative disorders (38, 39).

To the best of our knowledge, this study is the first investigating the longitudinal effect of NAC in the treatment of patients with chronic mTBI using rs-fMRI imaging. We collected rs-fMRI data from patients with chronic mTBI at baseline and after 3 months follow-up. Also, multiple rs-fMRI metrics were measured for each individual including fALFF, DC, and FCS. Neuropsychological tests were performed at each time point to investigate cognitive performance changes after treatment with NAC. Quantifying the relationship between alteration in brain and cognitive functions can be used as predictive biomarkers for therapeutic response and assists in understanding the mechanism of mTBI and develop treatment strategies. We hypothesized that after 3 months of using NAC, rs-fMRI measurements would change associated with improvement in neurocognitive performance and brain recovery.

## Methods

### Participants

A total of 50 patients including 21 males (age: 42.2 ± 14.2 years) and 29 females (age: 49.6 ± 14.8 years) suffering from mTBI with chronic symptoms enrolled in this study after providing a written informed consent, approved by the institutional review board (40). Participants were recruited from local neurology offices and from the local community by self-referral. Exclusion criteria included if the patients had a history of other neurological disorders, significant medical illness, a current substance-use disorder, or current Diagnostic and Statistical Manual of Mental Disorders, 5th Edition (DSM-V) Axis I psychiatric illness. This study was registered on clinicaltrials.gov with the following identifier: NCT03241732. mTBI was defined according to the Mayo Classification System for Traumatic Brain Injury Severity, in which an injury was classified as mild if it met the following criteria: loss of consciousness <30 min, amnesia for <24 h, and no abnormal MRI findings (41). Enrolled patients had to report a history of one or more prior TBIs (one or multiple) meeting these criteria for mild TBI and have no structural injury to the brain such as a contusion, dura penetration, hematoma, or brainstem injury. They had to meet ICD-10 criteria for chronic mTBI (i.e., post-concussion syndrome) based upon symptoms that were the result of TBI and could include dysfunctionality such as cognitive problems, emotional problems (e.g., depression or anxiety), headache, dizziness, irritability, hypersensitivity to auditory or visual stimuli, balance problems, insomnia, or other subjective complaints specifically associated with the TBI. Also, patients had to report the chronic symptoms lasting for at least 6 months from the most recent TBI. Patients underwent neuroimaging at the center of MRI imaging at baseline and 3 months later in all the participants.

### NAC administration

After baseline assessment and imaging, participants were divided into two groups; One group included 24 patients who received 3 months of NAC, and another group included 25 patients assigned as a waitlist control (CN) group without employing any treatment. NAC was obtained from the Institutional Pharmacy (Cumberland Pharmaceuticals). Pharmaceutical grade NAC is an intravenous (IV) medication mostly used for the treatment of acetaminophen overdose. NAC was supplied as a sterile solution in vials containing 200 mg/mL acetylcysteine. NAC doses were prepared for each patient by the study nurse. The dose was 50 mg/kg mixed into approximately 200 mL of D5W infused over approximately 1 hour 1× per week. Participants also received 500 mg NAC tablets supplied by the Marcus Institute of Integrative Health and took 1 tablet 2× per day on all days that they do not receive the IV NAC (42). They received the NAC IV plus oral regimen for 3 months (12 IVs) and then returned for follow up imaging and evaluation.

### Neuropsychological assessment

Clinical assessment of mTBI subjects experiencing chronic symptoms included a battery of self-reported measures including the State-Trait Anxiety Inventory, Beck Depression Inventory, Profile of Mood Scale, Rivermead Post Concussion Symptoms Questionnaire (RPQ-3 and RPQ-13), the Epworth Sleepiness Scale, and two cognitive tests – the Forward and Backward Digit Span, and the Trail Making A and B tests. Clinical assessments were performed on the same day of the imaging study at both the baseline and follow-up time-points.

### MRI acquisition

All MRI scans were performed using a 3 T Siemens Biograph mMR Positron Emission Tomography-MR (mMR PET-MR) scanner with a 32-channel head coil. A structural T1-weighted was acquired to use during segmentation and registration steps of data preprocessing. MRI parameters for the anatomical T1-weighted sequence were as follows: repetition time = 1,600 msec, echo time = 2.46 msec, field of view (FOV) = 250 mm × 250 mm, matrix = 512 × 512, voxel size = 0.49 × 0.49, 176 slices with slice thickness = 1 mm.

Next, a resting-state BOLD scan was administered using an echo planar imaging (EPI) sequence using the imaging parameters including: FOV = 240 mm × 240 mm; voxel size = 3 mm × 3 mm × 4 mm; TR = 2,000.0 msec; TE = 30 msec; slice thickness = 4 mm; number of slices = 34; number of volumes = 180; and acquisition time = 366 s. During rs-fMRI, the participants were asked to close their eyes, and rest quietly without thinking about anything.

### Data processing

The rs-fMRI data was processed using Data Processing Assistant for Resting-State fMRI (DPARSF. V6.1_220101; http://rfmri.org/DPARSF) (43) including the steps as follow: discarding the first 10 volumes to ensure stable longitudinal magnetization; slice timing correction; head motion correction using six rigid body motion parameters; co-registration of the mean realigned EPI image with T1-weighted structural data and normalization of the to the EPI template in Montreal Neurological Institute (MNI) space with a resampling voxel size of 3 × 3 × 3 mm; removing the effect of micro head motion using Friston 24-parameter model the 24 parameters including 6 head motion parameters, 6 head motion parameters of the previous scan, and the 12 corresponding squared items (44); applying the temporal band-pass filter of 0.01–0.08 Hz to reduce the effect of low-frequency drifts and high-frequency respiratory and cardiac noises. Finally, the head motion was measured using frame-wise displacement (FD) and the exclusion criteria applied if participants had excessive head motion (>2.0 mm translation and/or 2.0° rotation). All data processing steps were limited within GM. Statistical parametric mapping 12^1^ was used to segment the brain to the GM for each participant. Then, the generated probabilistic map was binarized using fslmaths tools (cutoff = 0.2) to make the GM mask (45).

Multiple rs-fMRI metrics were measured including fALFF, DC, and FCS. fALFF was measured as follows: for each participant, spatial smoothing [Gaussian kernel of full-width half maximum (FWHM) = 6 mm] was performed (7, 46). Then, with the FFT, the time courses of rs-fMRI signal were converted to frequency domain, and the square root of the power spectrum was measured and averaged across the 0.01–0.08 Hz domain. Then, voxel-wise fALFF was measured as the ratio of power in low-frequency band (0.01–0.08 Hz) to the power of the entire frequency range (0–0.25 Hz). While ALFF describes the local spontaneous brain activity across the whole brain, by estimating the amplitude of neural activity in the low-frequency range (0.01–0.08 Hz), fALFF is a normalized derivation of ALFF representing the ratio of low-frequency range amplitudes (0.01–0.08 Hz) relative to the entire frequency range (e.g., 0–0.25 if TR = 2 s) amplitudes. To ensure standardization, for each participant, the fALFF of each voxel was transformed to *z*-scores using Fisher’s *z*-transform, and zfALFF maps were obtained (47).

Likewise, the DC based on the concept of graph theory was measured by computing Pearson correlation coefficients between time courses of each pair of voxels and generating a correlation matrix. Further, to remove the weak correlations that might be induced by noise, a threshold of *r* > 0.25 was used to obtain the undirected adjacency matrix. Then, for each voxel, the DC was calculated as the sum the connections between this voxel with other voxels. For standardization purpose, the weighted DC was transformed to *z*-scores using Fisher’s *z*-transform. Finally, the zDC map was smoothed with an isotropic 6 mm FWHM Gaussian kernel (48).

Finally, the voxel-wise whole-brain FCS maps were measured by estimating Pearson’s correlations between the time series of any pairs of brain voxels within the GM mask. Then, for a given voxel i, FC was measured and the FCS maps were smoothed with a 6 mm FWHM Gaussian kernel (21, 49).

### Statistical analysis

In order to evaluate the distribution of gender and age within and between patient groups, a chi-square and two-sample *t*-test were employed, respectively. A value of *p* of ≤0.05 was considered as statistically significant. Furthermore, to identify the pattern of group differences (NAC versus CN) over time (baseline versus follow-up), two-way mixed-effect analysis of covariance (ANOVA) was performed for each rs-fMRI metric using the statistical analysis module in the Data Processing & Analysis of Brain Imaging (DPABI- V6.1_220101 toolbox; http://rfmri.org/dpabi) (43). The clusters identified significant by the group-by-time interaction were selected as the regions of interests (ROIs). Next, for each rs-fMRI metric, the mean values were extracted over the mask of ROIs. To test the direction of the main effect of group, post hoc two-sample *t*-test was used to detect rs-fMRI differences between NAC and CN groups at baseline and follow-up. Also, in order to explore the effect of time, post hoc paired *t*-test was employed to detect baseline to follow-up changes in CN and NAC groups, separately. Gaussian random field (GRF) theory was employed for correction of multiple comparisons (voxel significance *p* < 0.05, cluster significance *p* < 0.05). Age, gender, and mean FD were considered as covariates to eliminate interferences.

Pearson partial correlation analysis was employed to evaluate the association between longitudinal changes of rs-fMRI metrics in ROIs and neuropsychological test scores while adjusting the effect of age and gender (ΔfALFF = fALFF_follow-up_ − fALFF_baseline_; ΔDC = DC_follow-up_ − DC_baseline_; ΔFCS = FCS_follow-up_ − FCS_baseline_; ΔClinical score = Clinical score_follow-up_ − Clinical score_baseline_). The value of *p* <0.05 was considered as significant level. The name of the brain regions was recorded based on the Automated Anatomical Labeling (AAL) atlas (50).

## Results

### Demographic and clinical data

Demographic statistical analysis showed no significant difference between the age of patients in CN and NAC groups (two-sample *t*-test; *t*-value = 1.1 value of *p* = 0.27), as well as no significant difference in the proportion of males and females within each group (chi-square test; *χ*^2^ = 0.21, value of *p* = 0.65). The detailed information of the patient demographics and is summarized in Table 1.

**Table 1:** Demographic of patients in CN and NAC groups.

Demographic	CN TBI (*n* = 25)	NAC TBI (*n* = 25)	Value of *p*	Statistic
Age(years), mean (SD)	48.92 (14.43)	44.25 (15.39)	0.27	*T* = 1.1
Sex (M/F)	11/14	10/15	0.64	*χ*^2^ = 0.21
Injury-to-imaging interval (95% lower CI – 95% upper CI) (months)	23–37	24–35	–	–
Single concussion vs. multiple (single: multiple)	9:16	8:17	–	–

CN, control; NAC, N-acetyl cysteine; SD, standard deviation; M, male; F, female; *χ*^2^, statistic obtained chi-square test; *T*, statistic obtained by two-sample *t*-test; CI, confidence interval.

### rs-fMRI metrics analysis

#### Group-by-time interactions

**Figure 1:**
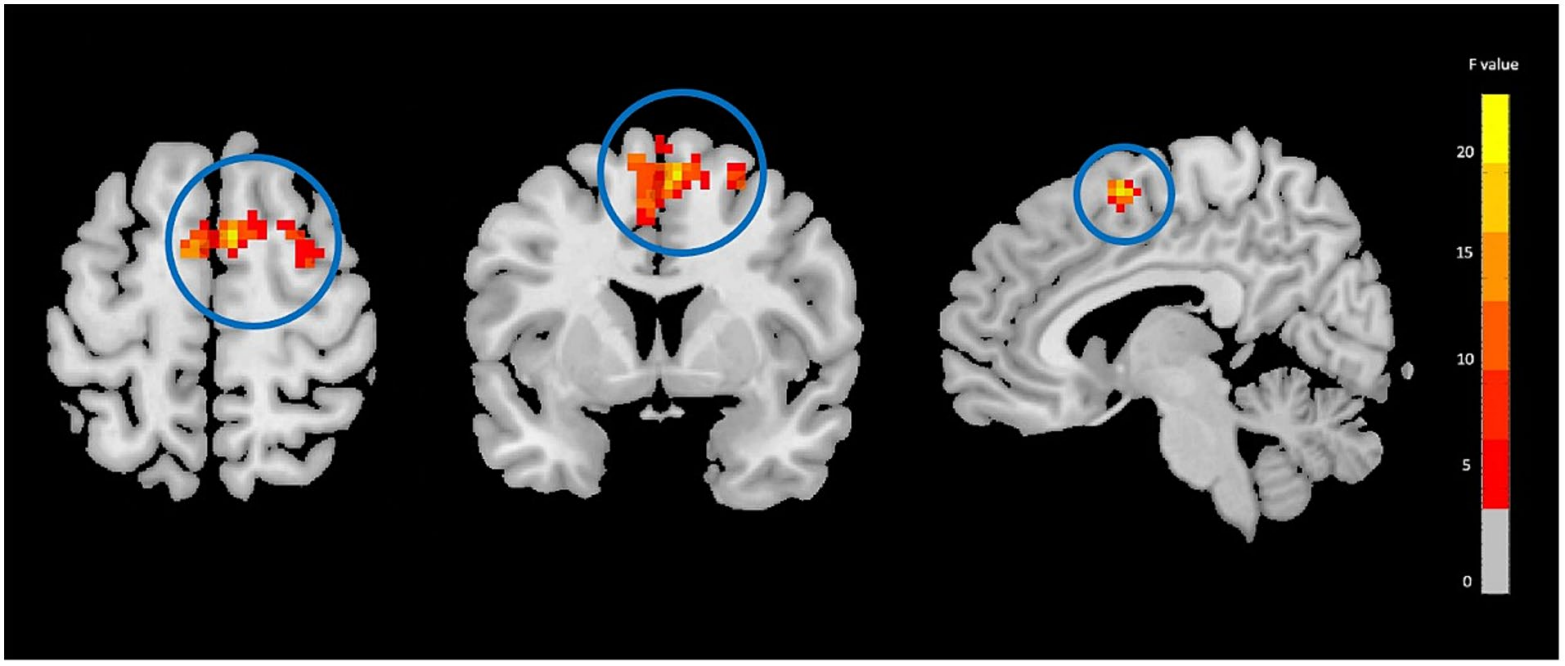
Fractional amplitude of low frequency fluctuations (fALFF) analysis showing significant group-by-time interaction effect brain regions between NAC (*n* = 25) and CN (*n* = 25) groups after 3 months follow-up including the right supplementary motor area in the peak of the cluster; GRF-corrected, voxel-level *p* < 0.05, cluster-level *p* < 0.05; NAC, NAC, N-acetyl cysteine; CN, controls. The range of *F*-values is represented by the color bar. *The right side of the image as displayed is the right side of the person.

**Figure 2:**
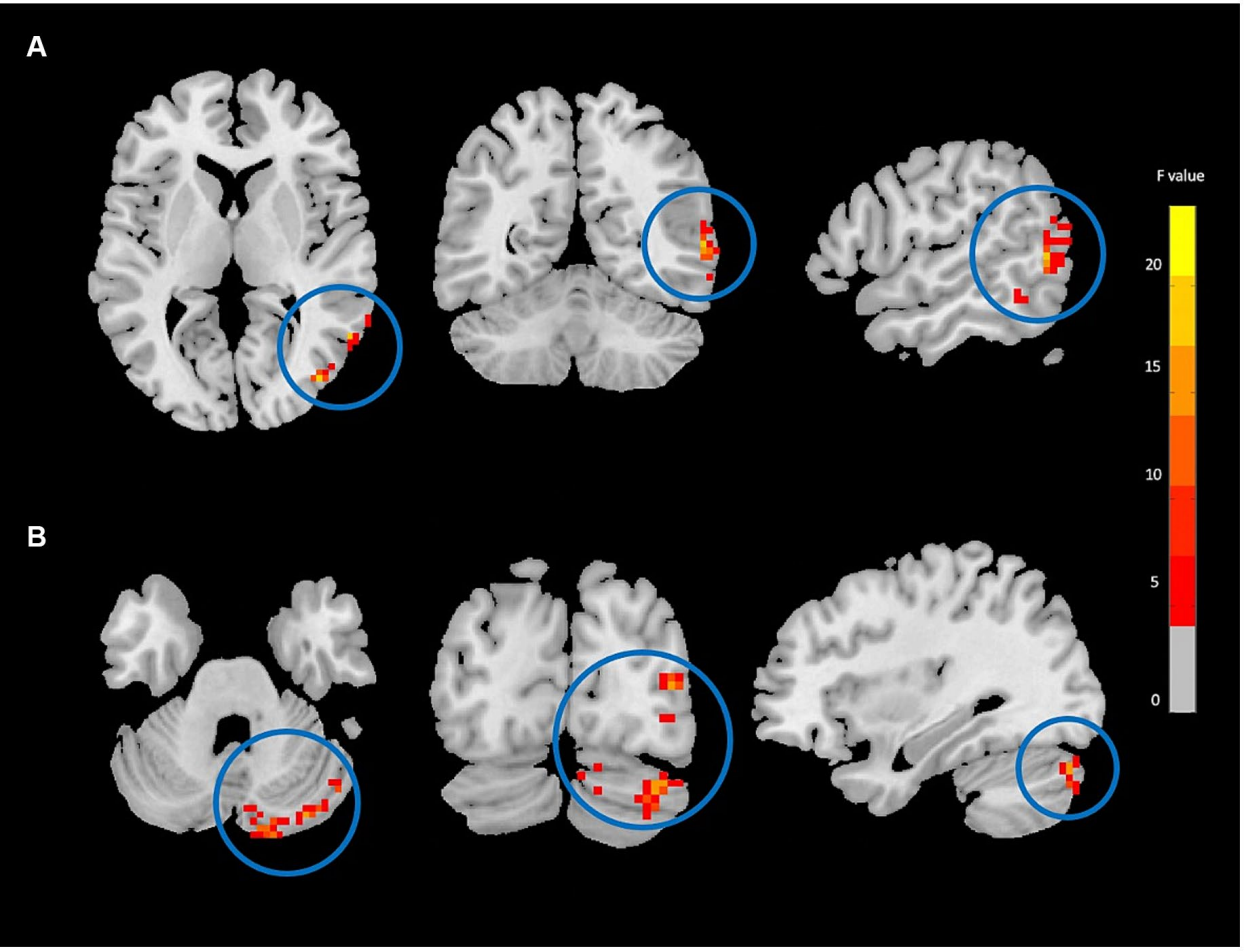
Degree centrality (DC) analysis showing significant group-by-time interaction effect brain regions between NAC (*n* = 25) and CN (*n* = 25) groups after 3 months follow-up including the right middle temporal gyrus **(A)**, and right cerebellum_crus1 **(B)** in the peak of the clusters; GRF-corrected, voxel-level *p* < 0.05, cluster-level *p* < 0.05; NAC, NAC, N-acetyl cysteine; CN, controls. The range of *F*-values is represented by the color bar. *The right side of the image as displayed is the right side of the person.

**Figure 3:**
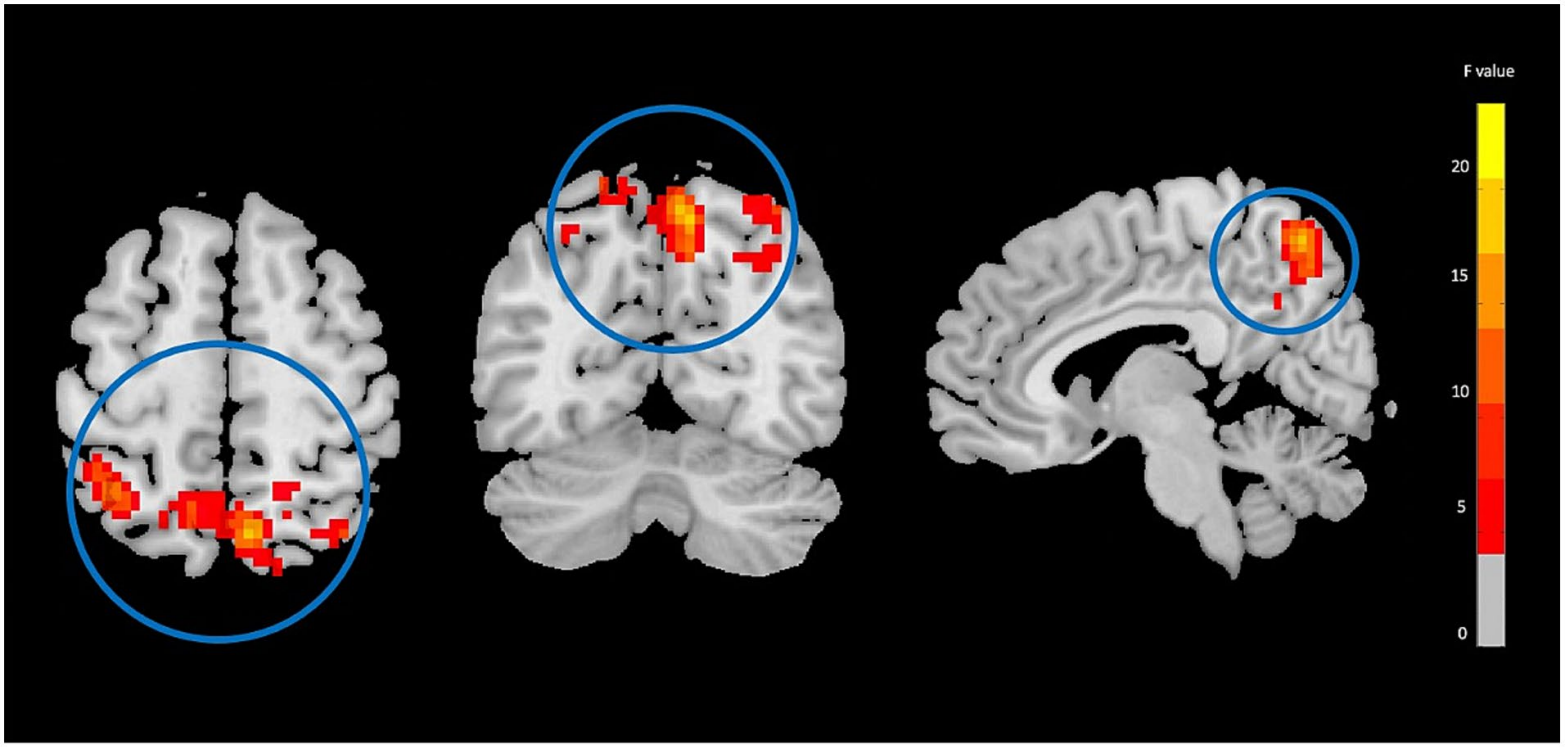
Functional connectivity strength (FCS) analysis showing significant group-by-time interaction effect brain regions between NAC (*n* = 25) and CN (*n* = 25) groups after 3 months follow-up including the right precuneus in the peak of the cluster; GRF-corrected, voxel-level *p* < 0.05, cluster-level *p* < 0.05; NAC, NAC, N-acetyl cysteine; CN, controls. The range of *F*-values is represented by the color bar. *The right side of the image as displayed is the right side of the person.

The two-way mixed-effect ANOVA results revealed significant group-by-time interactions. For fALFF analysis, we found a cluster located in the right supplementary motor area (rSMA) (F-score = 22.61, voxel significance *p* < 0.05, cluster significance *p* < 0.05); for DC analysis, two clusters located in the right middle temporal gyrus (rMTG) (F-score = 18.08, voxel significance *p* < 0.05, cluster significance *p* < 0.05) and right cerebellum_crus1 (rCerebellum) (F-score = 15.76, voxel significance *p* < 0.05, cluster significance *p* < 0.05); and for FCS analysis, a cluster located in the right precuneus (rPrecuneus) (*F*-score = 14.19, voxel significance *p* < 0.05, cluster significance *p* < 0.05)as significant ROIs. Figures 1–3 show the brain maps of the results of the group-by-time interaction analysis for each rs-fMRI metric over 3 months longitudinal study. Also, these results are represented in Table 2.

**Table 2:** Significant differences of group-by-time interaction effects between CN and NAC groups from baseline to follow-up for different rs-fMRI metrics.

Brain region	Voxels	Peak MNI coordinate (*x*, *y*, *z*)	*F*-score
**fALFF**			
Supp_Motor_Area_R	266	6 6 60	22.61
**DC**			
Temporal_Mid_R	130	57–57 6	18.08
Cerebellum_Crus1_R	93	33–81 −30	15.76
**FCS**			
Precuneus_R	590	6–66 57	14.19

CN, control; NAC, N-acetyl cysteine; *x*, *y*, *z*, coordinates of primary peak locations in the space of MNI; MNI, Montreal Neurological Institute; fALFF, fractional amplitude of low-frequency fluctuations; DC, degree centrality; FCS, functional connectivity strength; R, right; F, statistical value of peak voxel.

#### Post hoc analysis

Two-sample *t*-test comparing treatment groups on mean *z*-scores in the clusters identified in group-by-time interactions showed significant greater fALFF at baseline (value of *p* = 0.009), and lower fALFF at follow-up (value of *p* <0.001) in the rSMA in NAC compared to CN group. DC analysis showed greater DC at baseline (value of *p* <0.001), and lower DC at follow-up (value of *p* = 0.015) in the rMTG in NAC compared to CN group. Also, lower DC at follow-up (value of *p* = 0.001) in the rCerebellum were found in NAC compared to CN group. From FCS analysis, we found greater FCS at baseline (value of *p* = 0.032) in the rPrecuneus in NAC compared to CN group.

**Figure 4:**
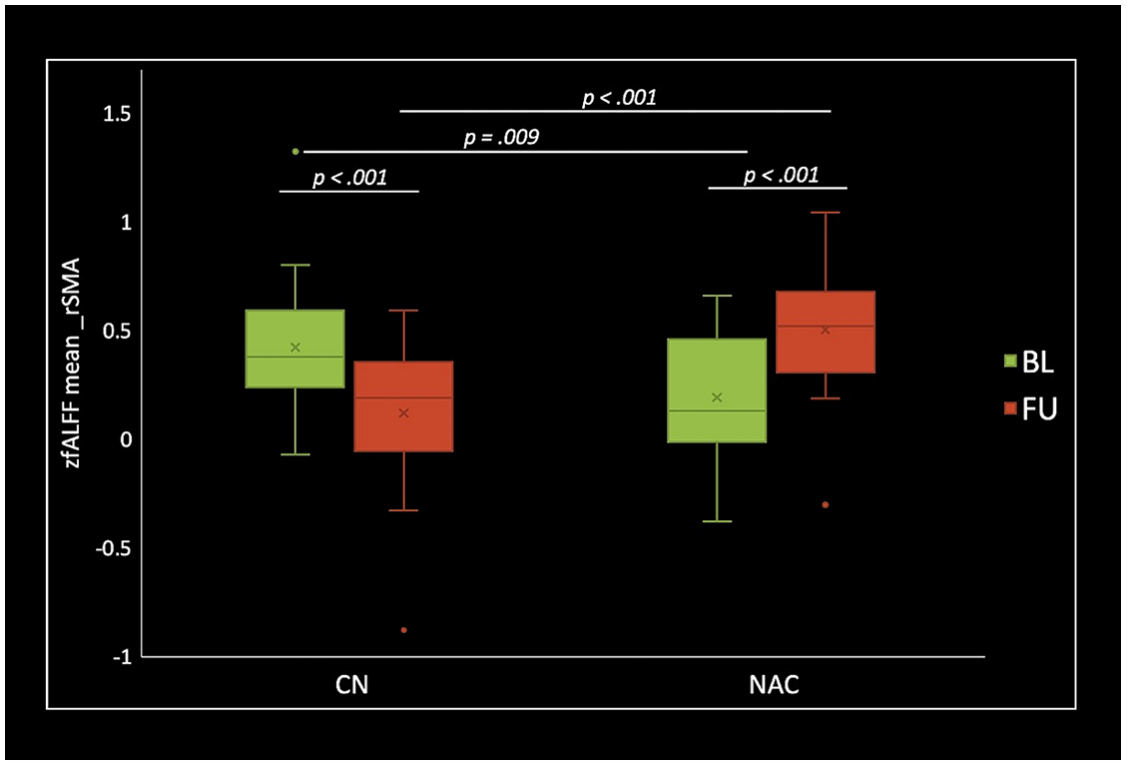
Box plots of mean fALFF *z*-scores in the right supplementary motor area (rSMA), post hoc paired *t*-test, and post hoc two-sample *t*-test among CN (*n* = 25) and NAC (*n* = 25) groups between baseline (BL) and 3 months follow-up (FU). fALFF, fractional amplitude of low frequency fluctuations; CN, controls; NAC, N-acetyl cysteine.

**Figure 5:**
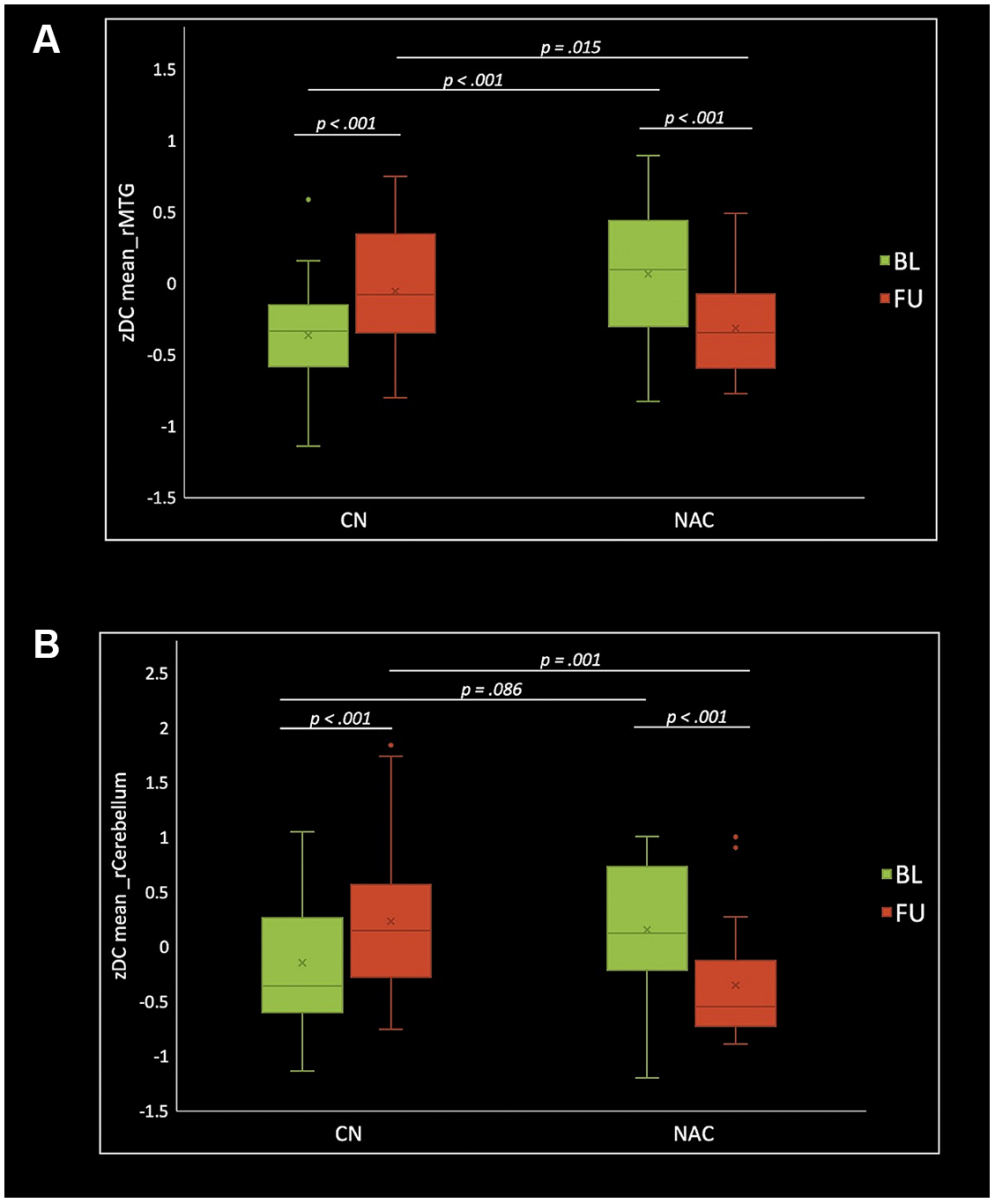
Box plots of mean DC *z*-scores in the right middle temporal gyrus **(A)** and right cerebellum_crus1 **(B)**, post hoc paired *t*-test, and post hoc two-sample *t*-test among CN (*n* = 25) and NAC (*n* = 25) groups between baseline (BL) and 3 months follow-up (FU). DC, degree centrality; CN, controls; NAC, N-acetyl cysteine.

**Figure 6:**
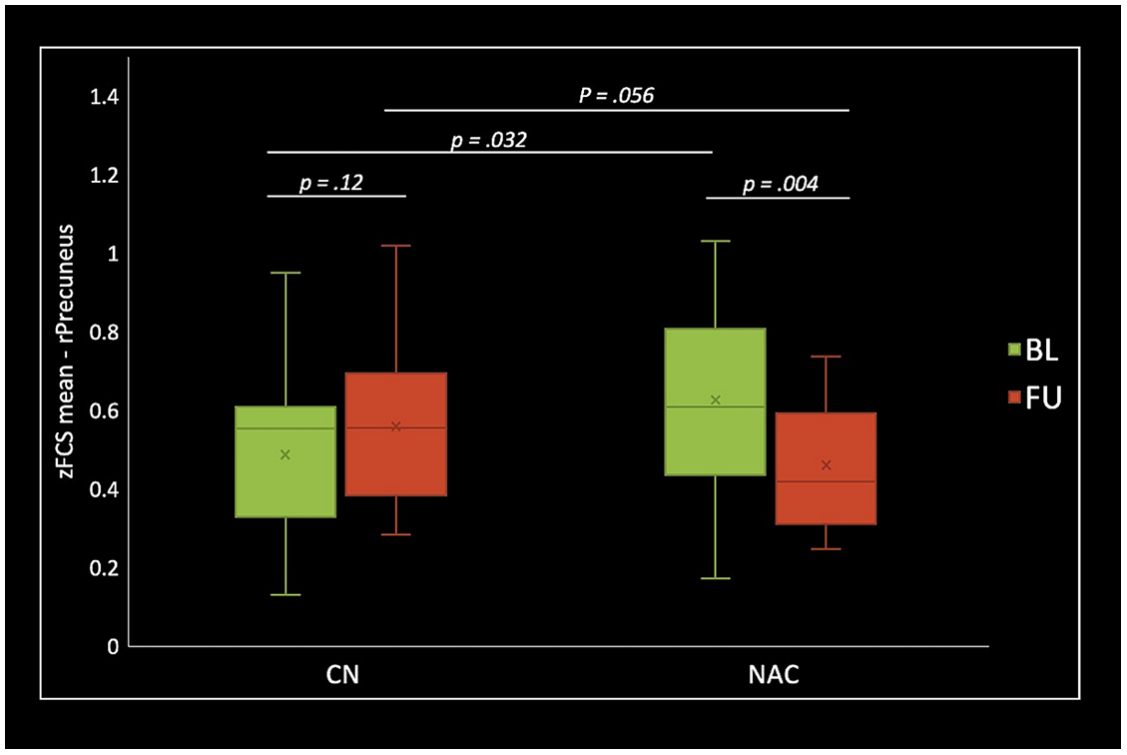
Box plots of mean FCS *z*-scores in the right precuneus (rPrecuenus), post hoc paired *t*-test, and post hoc two-sample *t*-test among CN (*n* = 25) and NAC (*n* = 25) groups between baseline (BL) and 3 months follow-up (FU). FCS, functional connectivity strength; *z*, *z*-score; CN, controls; NAC, N-acetyl cysteine.

Paired *t*-test examining within-group change over time showed significantly increased fALFF in the NAC group (value of *p* <0.001), and decreased fALFF in CN group (value of *p* <0.001) in the rSMA at follow-up compared to baseline. Likewise, mean DC values decreased in NAC group (value of *p* <0.001), and increased in CN group (value of *p* <0.001) in the rMTG; as well as decreased in NAC group (value of *p* <0.001), and increased in CN group in the rCerebellum at follow-up compared to baseline. Additionally, FCS analysis showed decreased FCS values in NAC group (value of *p* = 0.004) after treatment compared to baseline. Figures 4–6 show the box plots and visual representations of the post hoc analysis for each rs-fMRI metric among CN and NAC groups and between baseline and follow-up timepoints.

#### Correlation analysis

**Figure 7:**
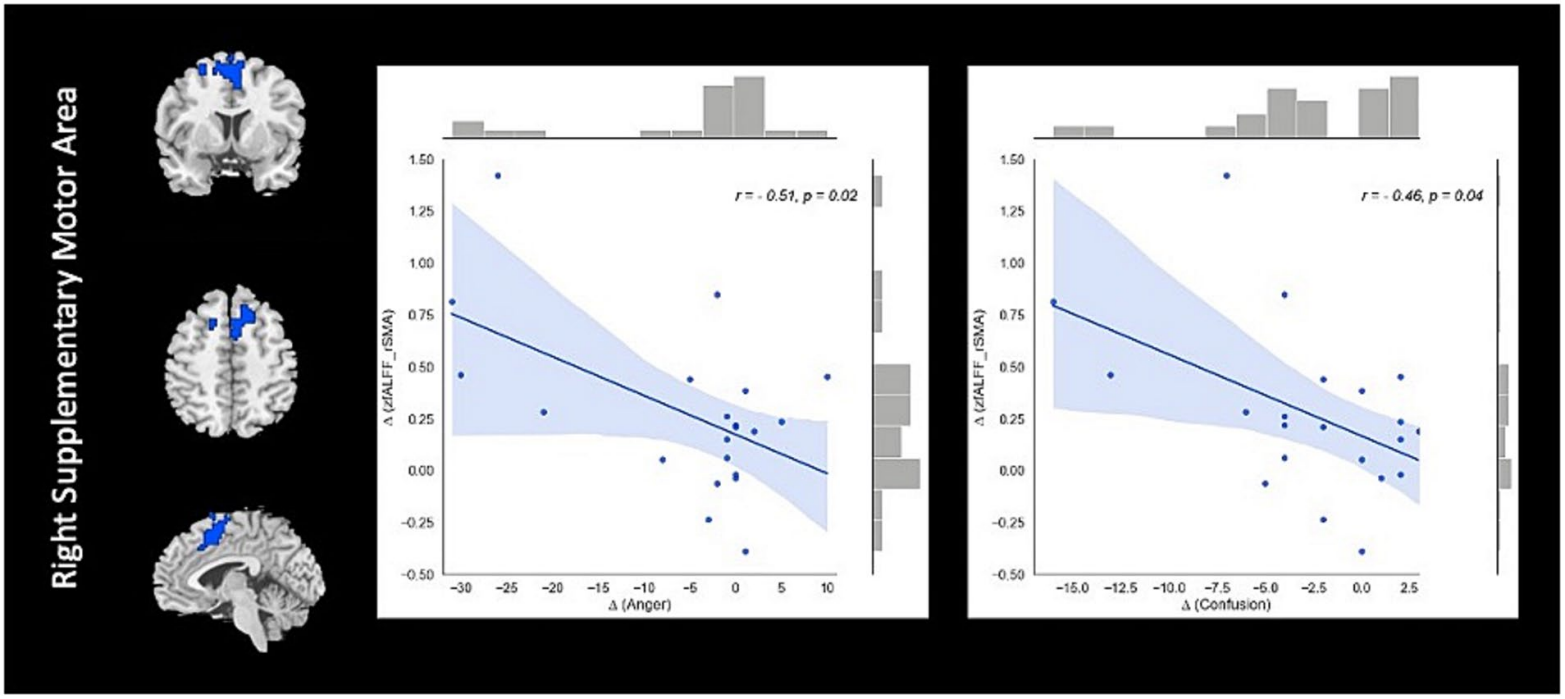
The correlation between longitudinal fALFF and clinical variable changes over 3 months treatment in NAC (*n* = 25) group in the significant group-by-time interaction region (*p* < 0.05). fALFF, fractional amplitude of low frequency fluctuations; rSMA, right supplementary motor area; NAC, NAC, N-acetyl cysteine.

**Figure 8:**
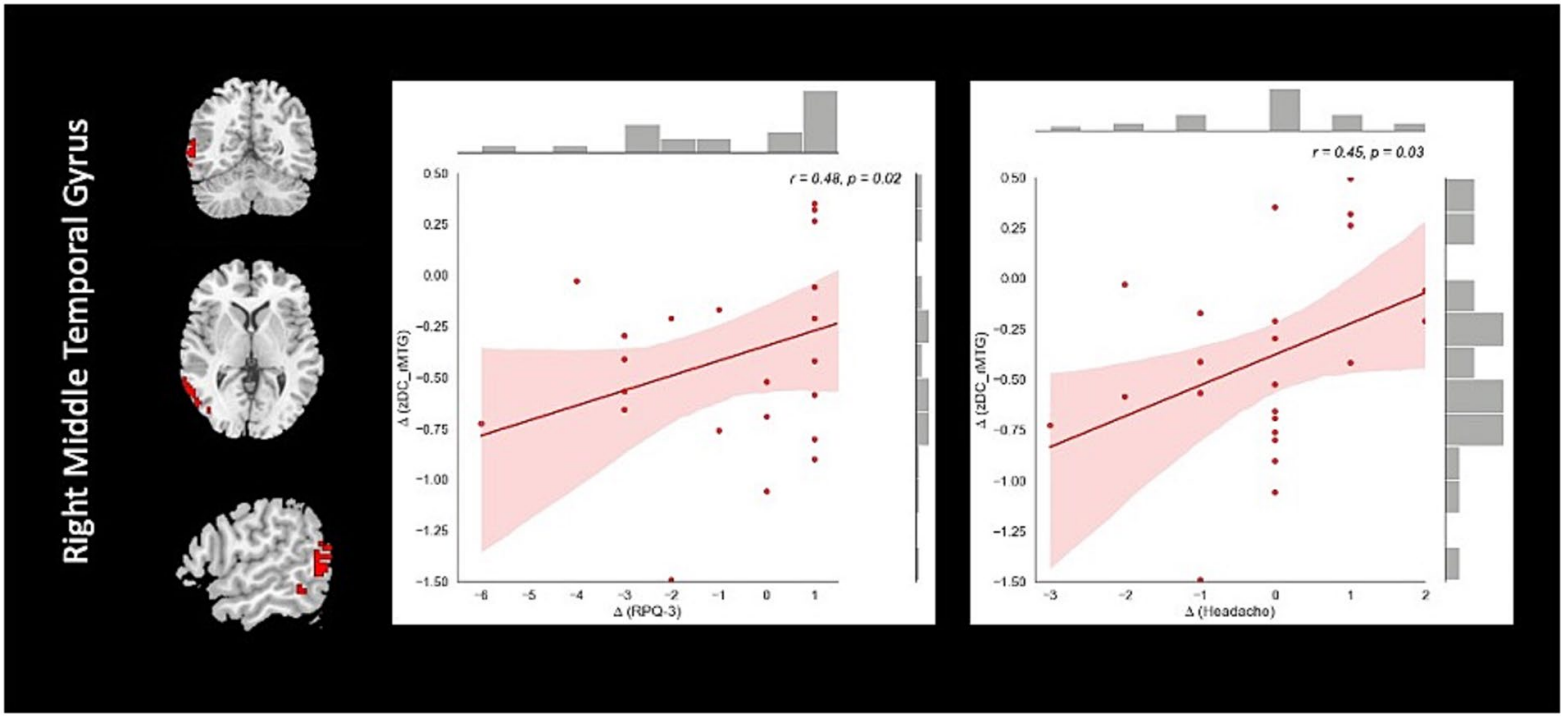
The correlation between longitudinal DC and clinical variable changes over 3 months treatment in NAC (*n* = 25) group in the significant group-by-time interaction region (*p* < 0.05). DC, degree centrality; rMTG, right middle temporal gyrus; RPQ, Rivermead post concussion symptoms questionnaire; NAC, N-acetyl cysteine.

**Figure 9:**
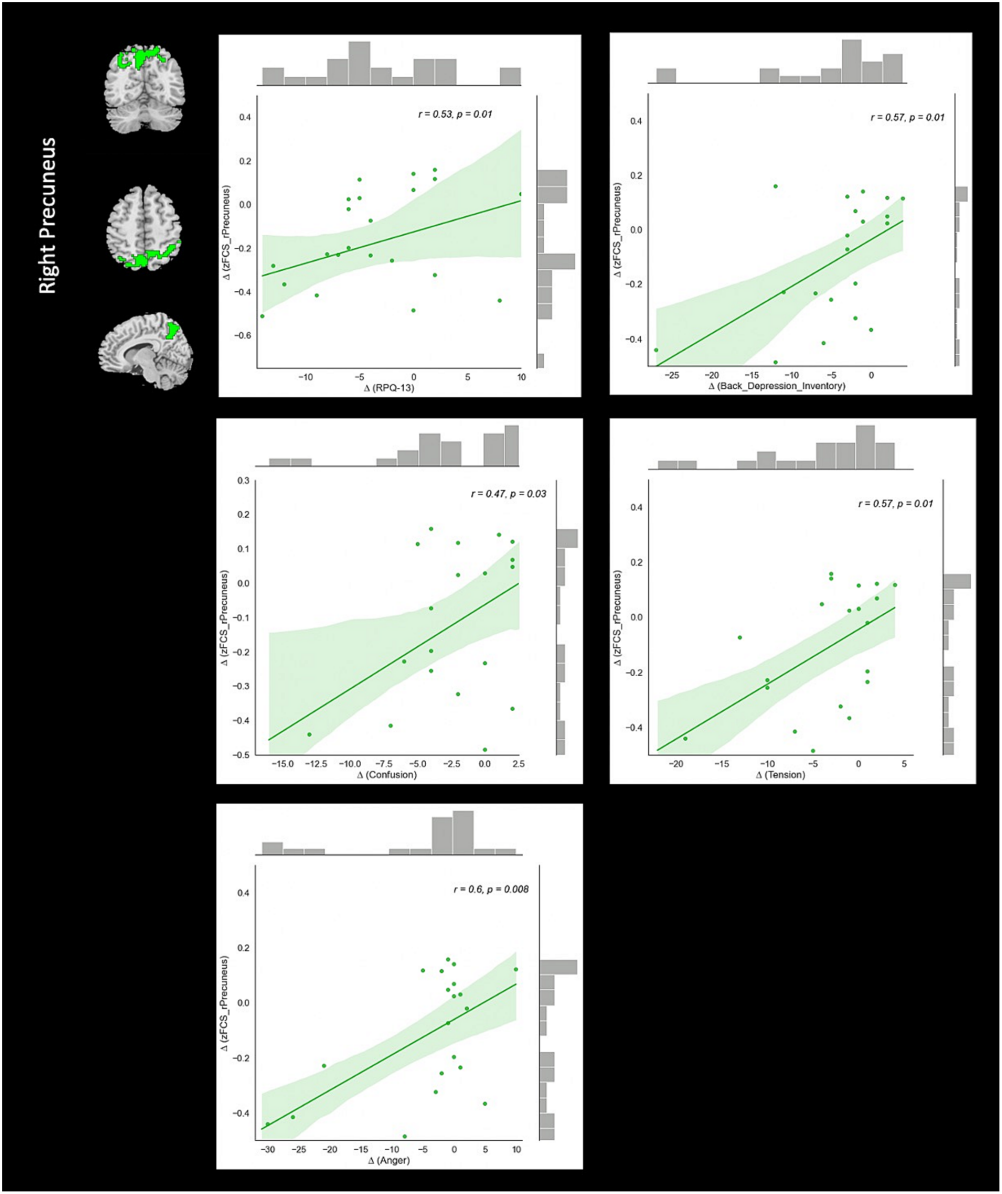
The correlation between longitudinal FCS and clinical variable changes over 3 months treatment in NAC (*n* = 25) group in the significant group-by-time interaction region (*p* < 0.05). FCS, functional connectivity strength; rPrecuneus, right precuneus; RPQ, Rivermead post concussion symptoms questionnaire; NAC, N-acetyl cysteine.

Correlation analysis revealed significant negative association between changes in fALFF in rSMA and anger (*r* = −0.51, value of *p* = 0.023), and confusion (*r* = −0.46, value of *p* = 0.046) clinical scores from baseline to follow-up timepoints in NAC group. Also, significant positive correlation between changes in DC in the rMTG and RPQ-3 (*r* = 0.48, value of *p* = 0.025), and headache (*r* = 0.45, value of *p* = 0.037) from baseline to follow-up timepoints in NAC group. Furthermore, we found significant positive correlation between alteration of FCS in the rPrecuneus and RPQ-13 (*r* = 0.53, value of *p* = 0.013), back depression inventory (*r* = 0.56, value of *p* = 0.011), tension (*r* = 0.57, value of *p* = 0.011), anger (*r* = 0.59, value of *p* = 0.008), and confusion (*r* = 0.47, value of *p* = 0.038) in NAC group. No significant association was found between changes in rs-fMRI measurements and clinical scores in CN group. The results of the correlation analysis are summarized in Table 3. Also, the plots of the significant correlations between alteration of rs-fMRI measurements and clinical variables in the clusters found significant by the group-by-time interaction analysis are represented in Figures 7–9.

**Table 3:** The correlation between the longitudinal rs-fMRI measurements and clinical variables changes in NAC group in the significant group-by-time interactions regions.

Brain region	Clinical test	Pearson’s *r*	Value of *p*
**fALFF**			
Supp_Motor_Area_R	Anger	−0.51	0.02
	Confusion	−0.46	0.04
**DC**			
Temporal_Mid_R	RPQ-3	0.48	0.02
	Headache	0.45	0.03
**FCS**			
Precuneus_R	RPQ-13	0.53	0.01
	Back Depression Inventory	0.56	0.01
	Tension	0.57	0.01
	Anger	0.59	0.008
	Confusion	0.47	0.03

rs-fMRI, resting-state functional magnetic resonance imaging; NAC, N-acetyl cysteine; fALFF, fractional amplitude of low-frequency fluctuations; DC, degree centrality; FCS, functional connectivity strength; R, right; RPQ, Rivermead post concussion symptoms questionnaire.

## Discussion

To our knowledge, this is the first study to investigate the longitudinal effect of NAC in patients with chronic mTBI using rs-fMRI imaging and neuropsychological assessment. Overall, there is a lack of research providing the evidence of examining pharmacological treatments for mTBI patients. We investigated the association between altered neuronal activity at rest at three levels including fALFF, DC, and FCS and cognitive performance after 3 months treatment with NAC. Two groups of patients were compared at baseline and 3 months follow-up including CN and NAC groups employing the mixed-effect ANOVA analysis. We hypothesized that different resting-state measurements capture different aspects of neuropathology and hence, providing complementary information underlying chronic mTBI. Using this approach, we found support for our hypothesis of alteration of rs-fMRI activity and cognitive recovery over 3 months treatment with NAC. Previous rs-fMRI studies have detected both reduction and increase of resting-state measurements in mTBI that could be attributed to several reasons, including the time of the study after brain injury, severity of the diseases, sample size, and heterogenous population phenotypes (22, 45).

### Effect of NAC on rs-fMRI measurement and cognitive performance

The pattern of fALFF, DC, and FCS observed in the NAC patients changed in a different way compared to the CN, and were accompanied by improvement in clinical cognitive function, providing evidence toward understanding the neuronal mechanism of chronic mTBI. fALFF analysis revealed alterations in opposite directions comparing between the CN and NAC groups over 3 months. The NAC group showed an increase of fALFF in rSMA that was negatively correlated with changes in cognitive variables including anger and confusion test scores. This finding highlights the clinical significance of fALFF change that could represent an important mechanism of action underlying NAC effects in neurobehavioral recovery in chronic mTBI patients. SMA as a part of superior frontal cortex (SFC) and default mode network (DMN) has been shown to be linked with cognitive processing and volitional aspect of motor planning in self-initiated movement (51). Numerous studies have shown that the frontal lobe is particularly at risk of contusion in moderate to severe brain injury (52). Additionally, it has been demonstrated that the frontal regions play roles in the affective and cognitive modulation of pain. As such, abnormal structure and function in individuals with different types of headaches and chronic pain is associated with the cognitive processing of pain (53). A recent study revealed that SFC as the subset of Brodmann area 10 (BA 10) is implicated in various integrative roles, including behavior control, movement planning, abstract reasoning, and modulation of cortical and subcortical nociceptive pathways (54). Given the pattern of reduction of fALFF in the rSMA in CN group and its increase in the NAC group over 3 months follow-up, we speculate that NAC can modulate the neuronal activity mechanism of this area in chronic mTBI patients that results in better cognitive and motor task performance. This is in line with the findings of a recent study demonstrating lower fALFF values in chronic mTBI patients with high symptom severity that may gradually increase to higher values close to healthy subjects following patient recovery. They also showed that the changes in fALFF are more prominent in specific brain networks including motor and visual networks, and this is associated with visuo-motor and cognitive dysfunction in mTBI patients (22).

Further, we found significant group-by-time interaction regions of DC analysis located in the rMTG and rCerebellum. After 3 months, the NAC group compared to the CNs demonstrated a significant decrease of DC in the rMTG and rCerebellum. The decreased DC in the rMTG was significantly correlated with decreased neuropsychological test scores including headache and RPQ-3 in the NAC group over 3 months follow-up. DC has been known to characterize node centrality in a network analysis. The higher the DC of a node, the more important that node in the network.

Medial temporal lobe pathology has been shown to play a significant role in a variety of neuropsychiatric and neurodegenerative disorders (55). Recent neuropsychological and neuroimaging literature suggested the vulnerability of the MTG in various aspects of working memory and emotional cognitive perception (56). Specially, frontotemporal network and brain activity dysfunction in this area has been known to be a target of mTBI which is mainly responsible for cognitive impairment in these patients (57, 58). A prior study showed increased functional connectivity between subnetwork nodes in the DMN including the temporal cortex areas in patients with chronic mTBI after 1 year post-injury that was linked with working memory and cognitive dysfunction in those patients (59). Our results are in agreement with the findings of a recent study revealing that the migraine-like headache is one of the most common phenotypes of mTBI that is associated with FC alteration, particularly in the brain regions located in the DMN, motor network, visual network, fronto-parietal network, dorsal and ventral attention network. They also suggested that the alteration in brain connectivity between these networks may shift the balance between network integration and segregation at the whole-brain level, necessitating the effective treatment strategies achieving the efficient neuronal communication (60). Additionally, alteration in brain function in the cerebellum has been shown to be linked with movement control, sensory perception, micro-motor coordination, and gait changes in patients with mTBI after injury (61). A recent study incorporated multi-modal analysis of rs-fMRI in mTBI patients and detected cerebellar-temporal lobe circuit dysfunction that was associated with abnormal motor and executive function in the patients (57).

From the brain FC perspective, we detected significant reduction of FCS in the rPrecuneus in patients under treatment with NAC that was positively correlated with RPQ-13, back depression inventory, tension, anger, and confusion test scores. Precuneus is the portion of superior parietal cortex and has been shown to be vulnerable to be impaired in brain injuries. For instance, a recent rs-fMRI study on the veterans with mTBI in the chronic stage demonstrated an increase of FC in several brain regions including the precuneus in patients compared to controls. They reported that this finding may reflect functional reorganization through neuroplasticity and compensation mechanisms following microstructural damage after post-injury (62). Additionally, Iraji et al. in a rs-fMRI independent component analysis (ICA) showed increased of FC in the precuneus in mTBI patients compared to healthy controls suggesting its implication in memory problems and the brain trying to recruit brain networks for recovery after concussion (63).

In line with this, Shumskaya et al. demonstrated increased FC within the right frontoparietal network in patients with acute mTBI compared to healthy subjects implying increased awareness to external environment, as well as excessive cognitive fatigue after post-injury (64). This study translated these findings into a real-world clinical setting indicating the engagement of precuneus connectivity in the presence of post-concussive symptoms that can be linked with emotional and cognitive recovery mechanism following treatment with the medication.

### Mechanism of actions of NAC and its effect on cognition

There is a significant literature on NAC as a neuroprotective agent in preclinical models of peripheral and central nervous injury. Also, a lot of studies have examined the treatment effects of NAC in animal models and human data reporting cognitive improvement in both preclinical and clinical studies in neurodegenerative and neuropsychiatric disease including AD (65), PD (66), stroke (67), depressive and anxiety disorder (68, 69), and mTBI (70). Over the past few decades, experimental research in brain injury models has emphasized the contribution of free radical-induced oxidative damage following secondary CNS injury. Of note, several therapeutic approaches including pharmacological antioxidants have been examined to validate their neuroprotective effects in preclinical experimental head injury models. Among them, NAC has been proved to be an effective antioxidant agent that positively impacts global functioning and clinical neurobehavioral symptoms (29). The results of this study are in line with the literature demonstrating the neuroprotective efficacy of NAC in ameliorating biochemical and neuroinflammation and treating spine-related neurodegenerative diseases including mTBI. A prior study evaluated the treatment effects of NAC on symptomatic U.S. service members who were exposed to significant ordnance blast and met the criteria for mTBI. Neuropsychological tests were also administered to assess the executive function after 7 days post-treatment. They have found that NAC had significant impact on neuropsychological test results, some mTBI symptoms, and symptom resolution by day seven of treatment. Particularly, confusion resolved after early treatment as well as Trail Making A and B were restored to normal within seven days after blast-induced mTBI. They proposed that post-treatment with NAC can provide protection against neuronal death which is due to its antioxidant and anti-inflammatory properties (32). Moreover, a previous study examined the treatment effect of NAC in fetal inflammatory responses in neonatal brain injury that could be associated with long-term offspring cerebral injury. Using MRI imaging, they found maternal NAC therapeutic approach may influence brain micro-structure integrity and could be effective in human pregnancies followed by maternal/fetal inflammation (38). Furthermore, a recent study on patients with progressive multiple sclerosis (PMS) evaluated the anti-inflammatory effect of NAC as the percentage change of brain atrophy estimated by MRI measurements over 12 months among treatment and control groups. They showed that longer intervention with high dose of NAC targeting oxidative stress can moderate neurodegeneration in MS patients (39).

NAC can increase glutamate extracellular levels by activating neuronal cysteine-glutamate exchange system resulting in improvement of the cellular bases for regulation of motivation and memory within the nucleus accumbens (67, 71). Furthermore, the potential therapeutic efficacy of NAC has been examined in rodent models of mTBI. This study provided evidence confirming the efficient antioxidant activity of NAC in reversing and preventing cognitive abnormalities in mTBI patients. NAC acts as a precursor for glutathione which is an important intracellular antioxidant and prevents the damage induced by reactive oxygen species. It synthesizes within its target cells from amino acids, L-cysteine, L-glutamic acid and glycine and more importantly it is responsible for antioxidant activity of glutathione which eventually ameliorates the disruption of neurobehavioral and working memory in mTBI animal models (26).

Consistent with these findings, a previous study demonstrated that chronic NAC treatment reversed anxiety-like and depressive-like behaviors in neonatal clomipramine animal model of depression. They reported that antidepressant-like effects of NAC can modulate glutamatergic receptors, mitochondrial dysfunction, and astroglial glutamate exchanger improving cognitive deficits in a dose-dependent manner (72). In line with this, a recent study investigated the therapeutic effects of NAC administration in management of stress-related disorder in an animal model. They illustrated that the deleterious effects of chronic stress in the CNS are induced by glutamatergic hyperactivity, oxidative stress, increased inflammatory response, and glutathione depletion which can be reversed using NAC agent. Lastly, they concluded that NAC is a beneficial treatment approach to resolve oxidative damage in mood and anxiety disorder, post-traumatic stress disorder, and other stress-related conditions (68). A prior study in an animal model of AD revealed the efficacy of NAC treatment in reversing social isolation induced by oxidative stress, γ-secretase activity, and increased Aβ-40 and Aβ-42 levels in AD model mice (65). Moreover, NAC treatment has also been assessed in a recent schizophrenia animal model study, demonstrating the restoring oxidative balance and prevention of impairment in declarative memory during prodromal stage of the disease (73). Additionally, Haber et al. in a rat model of mTBI incorporated NAC medication in the treatment plan and provided the evidence confirming the anti-inflammatory and antioxidant properties of NAC that can restore memory by modulation of glutamatergic neurotransmissions and reducing the level of interleukin 1β (IL-1β), tumor necrosis factor-α (TNF-α), and intercellular adhesion molecule-1 (ICAM-1). After NAC administration, it enters the brain through cysteine transporters. The proper dose of NAC may elevate extracellular glutamate levels in the hippocampus and restore memory in mTBI patients (74, 75). Given the NAC’s mechanism of action to ameliorate or prevent the cascade of pathological event after mTBI, we speculate that improved cognitive performance after treatment could be linked with the improved cellular basis for memory and regulation of motivation associate with the nucleus accumbens following NAC treatment. In fact, NAC may modulate disruption of memory and motivation via neural activation of cysteine-glutamate exchange that is enhanced by indirect effect of NAC on the metabolic glutamate receptors, mGluR2/3 and mGLuR5 (32, 76). Moreover, our results are consistent with the findings of studies representing the efficacy of NAC treatment as a neuroprotective agent in prevention of mitochondrial damage and loss of dendritic spines in hippocampal neurons based on animal models of ischemia-reperfusion brain injury, closed head trauma, cerebral stroke, and sensory nerve axotomy (67, 76, 77).

It has been shown that rapid and extensive production of free radicals and ROS after brain injury may cause disruption of the system. Additionally, the TBI-induced glutamate excitotoxicity promotes the intracellular ROS levels, which can be detoxified by mitochondrial manganese superoxide dismutase (MnSOD) or SOD2. The hypofunction of this antioxidant enzyme may lead to neurodegenerative disorders. A recent animal study has shown the neuromodulation effect of SOD2 that causes alteration in glutamatergic synaptic transmission in the hippocampus and improves memory deficits. Also, they have found oxidative stress followed by cellular damages in the brain that induced by the attenuated cellular SOD2 activity due to TBI (31). Moreover, it has been shown that increased level of ROS and pro-inflammatory cytokines in brain capillaries may result in blood brain barrio (BBB) permeability and promote the levels of neuroinflammation. Such increased oxidative stress within perivascular regions may lead to decreased hippocampus neurogenesis and long-lasting neurological impairment including memory dysfunction, depression, and anxiety (30).

Several studies have demonstrated that NAC has low bioavailability but can cross BBB when high doses of NAC are administrated. Additionally, an amide derivative of NAC known as N-acetylcysteine amide (NACA) has been shown with higher BBB permeability and easier penetration to mitochondria and other cellular constituents compared to NAC resulting in higher central nervous system bioavailability and stronger therapeutic properties for oxidative stress and neuroinflammation following TBI. However, no study has been done in humans using NACA. Both NAC and NACA are FDA approved to be being used in TBI patients (25, 70). Hence, further pre-clinical and clinical research are needed to establish better understanding of action and therapeutic efficiency of NACA in human TBI subjects.

Overall, the results of this study are in accordance with the literature revealing the potential neuroprotective effects of NAC on amelioration of cognitive and memory dysfunction in mTBI patients with chronic symptoms. Our findings support and extend the earlier findings of the pathophysiology of mTBI that engages multiple brain networks heterogeneously, indicating disruption of brain communication between the functional network modules. The hub organization in mTBI may interfere with a variety of integrative roles such as executive function, attention, working memory, and emotional processing (22, 78, 79). Given the potential therapeutic efficacy of NAC against mTBI, we postulate that a short-term treatment with NAC might be helpful to modulate functional reorganization and to facilitate neuroplasticity through recovery profile in patients with post-concussion symptoms promising the enhancement in neuronal compensatory mechanism and neurocognitive performance.

### Limitations and future work

This study has several limitations. First, considering the relatively small sample size, future studies need to include larger samples in both control and medication groups and acquire neuropsychological tests to estimate the behavioral performance after treatment. Second, potential inhomogeneities of the patient population in both CN and NAC groups might affect our results. Future experiments need to be more considerate including homogenous datasets in each group regarding the gender, age, severity of the disease, symptom types, and the time of study after brain injury. Third, a short follow-up data point was implemented in this study. Hence, our findings may not be generalized to long-term treatment with the medication. Therefore, longer periods and several follow-up time points are warranted to validate these findings. Lastly, further longitudinal studies could be designed using different doses of NAC and evaluate the persistency of the effect of the medication after treatment to define the optimum dose(s) that can be translated into preclinical and clinical settings.

## Conclusion

This study provided the evidence in understanding of pathophysiology of mTBI and the effect of NAC treatment using different rs-fMRI metrics. Also, we showed that 3 months treatment with NAC is associated with better cognitive performance in chronic mTBI. This study illustrated the circuitry involved in NAC treatment particularly in the brain networks including DMN, sensorimotor network, and emotional processing circuits. It appears that neuromodulation effect of NAC involves spontaneous brain activity at rest in different brain regions that is linked with clinical cognitive recovery according to the standard neuropsychological assessments. Future research is needed to confirm these findings using different medications, doses, and periods after treatment to provide novel therapeutic plans in mTBIs.

## Data availability statement

The raw data supporting the conclusions of this article will be made available by the authors, without undue reservation.

## Ethics statement

The studies involving humans were approved by Thomas Jefferson University institutional review board. The studies were conducted in accordance with the local legislation and institutional requirements. The participants provided their written informed consent to participate in this study. Written informed consent was obtained from the individual(s) for the publication of any potentially identifiable images or data included in this article.

## Author contributions

FV: Conceptualization, Methodology, Validation, Formal analysis, Investigation, Resources, Data curation, Writing - review & editing, Visualization, Supervision, Project administration. AN: Conceptualization, Methodology, Data curation, Writing - review & editing, Supervision, Project administration, Funding acquisition. MA: Methodology, Validation, Formal analysis, Investigation, Writing - review & editing. GZ: Resources, Project administration, Funding acquisition, Writing - review & editing. EN: Data curation, Writing - review & editing. CH: Data curation, Writing - review & editing. NW: Resources, Project administration, Funding acquisition Writing - review & editing. FM: Conceptualization, Methodology, Data curation, Writing - review & editing, Supervision, Project administration, Funding acquisition. DM: Conceptualization, Resources, Project administration, Funding acquisition, Writing - review & editing.

## Footnotes

^1^ https://www.fil.ion.ucl.ac.uk/spm/
